# Remarkable Migrations of a Pin and Needle through the Body of a Young Lady

**Published:** 1851-09

**Authors:** Napoleon B. Anderson

**Affiliations:** Louisville, Ky.


ART. IV.—*Remarkable migrations of a Pin and Needle through the Body of a Young Lady.* By NAPOLEON B. ANDERSON, M.D., of Louisville, Ky.

On the 20th of April, 1849, Miss Catherine M——, æt. 19 years, in a fit of laughter, accidentally swallowed a large brass pin and a medium sized needle. No pain attended the passage of these bodies into the stomach, nor was any felt until after the expiration of about the third week, at which time a warm, pricking sensation was first felt in the cardiac orifice of the stomach, which position it maintained for the space of three months, when it gradually changed, and seated itself in the lower lobe of the left lung. In this situation it remained for some nine months, without any disturbance to the organ of respiration in which it was felt, with the exception of occasional cough and slight hemoptysis. During this period, the pain gradually moved to the glenoid cavity of the scapula, and was experienced at the insertion of the deltoid muscle, in which situation considerable pain was the result of elevation or rotation of the arm. From this point it moved to the arm pit, when the arm had to be carried horizontally, and no elevation, rotation, adduction, or abduction could be performed without excruciating pain; the inner part of the arm turning very black, from the infiltration, I suppose, of blood into the surrounding parts. Pressure upon the parts, produced no material change in coloration, nor was there any unusual amount of sensation or numbness in any part of the discolored portion, with the exception of the region in which these foreign bodies were situated. The arm remained in this condition, with no material changes, until December, 1850, when the pain and uneasiness moving from the arm-pit, towards the articulation of the ulna and radius with the humerus, settled in the belly of the biceps flexor muscle, forming there a dark spot the size of a half dollar, and very sensitive to the touch. An emollient poultice was applied for twenty-four hours, when fluctuation indicated the use of the knife. A quantity of bloody pus was discharged, and the needle and pin were extracted from two different apertures, about half an inch apart. The pin was dark, but the needle was bright, and had undergone no material change. Alteratives were used, and in ten days from the extraction of the bodies, the lady had perfect use of her arm, and has continued to do so ever since.

During the period, from the swallowing of these substances until their removal, the constitution was not disturbed in the slightest degree, except the cough and hemoptysis spoken of; and this continued only as long as those articles were passing through the lungs, after which the symptoms disappeared. The lady underwent no treatment during their migration from the mouth to the arm, with the exception of a purge when she first swallowed the articles, and anodyne embrocations afterwards.

These pointed bodies appear to have traveled side by side over the entire route from the mouth to the point at which they were extracted, and must, in their course, have passed through the stomach, diaphragm, lung, pleura, among muscles and bloodvessels, before reaching the parts from which they were extracted. The points of each article presented at the incision made, and must, I suppose, have thus passed the entire distance.

July 8, 1851.

